# Effectiveness and safety of rituximab monotherapy versus conventional regimens for adult idiopathic membranous nephropathy: real-world retrospective study

**DOI:** 10.3389/fimmu.2025.1671251

**Published:** 2025-11-11

**Authors:** Jing Huang, Yingying Yang, Yongmei Wang, Shiyin Jiang, Ying Zhang, Shimin Zhao, Shuang Wang, Bing Chen, Gang Liu

**Affiliations:** 1 Department of Nephrology, The Second Hospital of Shandong University, Jinan, China; 2 Department of Neurology, The People’s Hospital of GaoTang, Liaocheng, China; 3 Department of Nephrology, Jinan Shizhong People’s Hospital, Jinan, China; 4 Department of Nephrology, Shandong Provincial Hospital, Jinan, China

**Keywords:** idiopathic membranous nephropathy, treatment regimens, clinical remission rate, anti-PLA2R antibody, rituximab

## Abstract

**Background:**

The 2021 Kidney Disease: Improving Global Outcomes (KDIGO) guidelines recommend immunotherapeutic regimens for idiopathic membranous nephropathy (IMN), including glucocorticoids (GC) with cyclophosphamide (CYC) or calcineurin inhibitors (CNIs), as well as biologics. However, the comparative effectiveness remains insufficiently explored. This study aimed to evaluate the effectiveness and safety of rituximab (RTX) versus conventional regimens.

**Methods:**

This study retrospectively included 310 IMN patients diagnosed with nephrotic syndrome (NS), who were divided into three groups: RTX group (n=62), glucocorticoid with cyclophosphamide (GC+CYC) group (n=124), and glucocorticoid with calcineurin inhibitor (GC+CNI) group (n=124). Treatment effectiveness and safety were assessed at the 12 months. The primary endpoint was clinical remission at 12 months. Secondary endpoints, included clinical remission rate, relapse rate, and safety and occurrence of adverse events(AEs)at 24 months.

**Results:**

At 12 months, 44/62 (71.0%) achieved clinical remission, with 18 (29.0%) achieving CR in the rituximab group,. In the GC+CYC group, 90/124 (72.6%) achieved clinical remission, 48 (38.7%) achieving CR. In the GC+CNI group, 97/124 (78.2%) achieved clinical remission, with 52 (41.9%) achieving CR. At 24 months, 33/35 (94.3%) achieved clinical remission, with 18 (51.4%) achieving CR in the RTX group. In the GC+CYC group, 102/115 (88.7%) achieved clinical remission, with 44 (38.3%) achieving CR. In the GC+CNI group, 98/114(86.0%) achieved clinical remission, with 47 (41.2%) achieving CR. The clinical and complete remission rates were significantly higher in the rituximab group than in the conventional treatment groups (clinical remission: 94.3% vs. 88.7% vs. 86.0%, *P* = 0.002; CR: 51.4% vs. 38.3% vs. 41.2%, *P* = 0.000). Logistic regression analysis revealed anti-phospholipase A2 receptor (PLA2R) antibody titer (OR = 0.998, *P* = 0.016) was identified as an independent risk factor for non-remission. The RTX group showed lower rates of overall AEs (27.4%), none of the AEs were severe.

**Conclusion:**

Rituximab demonstrated non-inferior clinical remission rates at 12 months compared to CYC and CNIs. Rituximab was also associated with lower relapse rates and better safety profile. These findings suggest that rituximab offers distinct advantages in maintaining long-term clinical remission and may be considered an effective treatment regimen for IMN patients at risk of disease progression.

## Introduction

Idiopathic membranous nephropathy (IMN) is a common cause of nephrotic syndrome (NS) in adults. Pathologically, IMN is characterized by subepithelial immune complex deposits, primarily composed of IgG and C3, accompanied by thickening of the glomerular basement membrane. Clinically, IMN is characterized by heavy proteinuria. If left untreated, patients with persistent heavy proteinuria are at risk of progressing to end-stage renal disease (ESRD) (1, 2). Therefore, proactive intervention and effective treatment for IMN patients are crucial for delaying disease progression.

In recent years, with the widespread use of biomarkers such as anti-phospholipase A2 receptor (PLA2R) antibodies (3–5), the diagnosis and treatment of IMN have increasingly shifted toward individualized and precision-based approaches. However, selecting the optimal treatment regimen remains a major topic of clinical debate. Currently, the main immunosuppressive treatment regimens include glucocorticoids (GC) (hereafter referred to as steroids) combined with the alkylating agent cyclophosphamide (CYC), GC combined with calcineurin inhibitors (CNIs), and rituximab (RTX) (6, 7). The differences in efficacy, safety, and long-term outcomes among these three treatment regimens urgently require systematic comparison based on large-scale clinical data.

In traditional treatment regimens, GC combined with an alkylating agent can inhibit the activation and proliferation of T and B cells, thereby reducing the production of autoantibodies. However, long-term use may carry risks such as bone marrow suppression, infection, and gonadal suppression (8, 9). CNIs, such as cyclosporine A and tacrolimus, primarily inhibit T-cell activation and proliferation. They can also affect renal hemodynamics and exert proteinuria-reducing effects by stabilizing podocyte structures. Nonetheless, the high relapse rate after discontinuation of this regimen and the nephrotoxicity associated with CNIs remain significant concerns (10). In contrast, RTX—a monoclonal antibody targeting CD20—reduces proteinuria by directly inducing B-cell apoptosis or by eliminating CD20-positive B cells through antibody-dependent cell-mediated cytotoxicity and complement-dependent cytotoxicity. Consequently, B-cell counts decline, and antibody production is reduced. RTX currently represents a promising new treatment option for a broader population of IMN patients (11, 12).

The 2021 Kidney Disease: Improving Global Outcomes (KDIGO) clinical practice guidelines (6) for glomerular diseases and the expert consensus (13) on the use of RTX in membranous nephropathy both emphasize individualized treatment based on risk stratification. These guidelines and the consensus recommend RTX or CYC combined with GC as initial treatment regimens for patients at intermediate to high risk. This study aimed to compare the effectiveness, safety, and target populations for various treatment regimens. The findings will provide evidence-based support for optimizing treatment strategies for IMN.

## Materials and methods

### Study participants

This study included 394 adult patients with NS who first visited the Department of Nephrology at Shandong Provincial Hospital, affiliated with Shandong First Medical University, between January 2015 and December 2023. All patients were diagnosed with IMN through renal biopsy. A total of 84 patients were excluded due to insufficient follow-up or failure to receive angiotensin system blockers for at least 3 months. Consequently, a total of 310 IMN patients were included in the final analysis.

Inclusion Criteria: (1) All patients underwent at least two separate 24-hour urine protein quantifications on different days, with each measurement exceeding 3.5 g/24 h and serum albumin levels below 30 g/L. (2) All patients had stable kidney function before treatment, defined as an estimated glomerular filtration rate (eGFR) ≥ 40 mL/min/1.73 m² or a 24-hour endogenous creatinine clearance rate > 40 mL/min/1.73 m². (3) All patients received angiotensin system blocker treatment for at least 3 months before treatment.

Exclusion Criteria: (1) Patients with irregular medication use or incomplete follow-up records were excluded. (2) Patients with secondary NS due to factors such as malignancy, heavy metal exposure, viral hepatitis, medication-related causes, metabolic diseases, or immune-mediated causes were excluded. (3) Patients with electron-dense deposits in subendothelial or mesangial areas observed on electron microscopy were also excluded.

**Figure 1:**
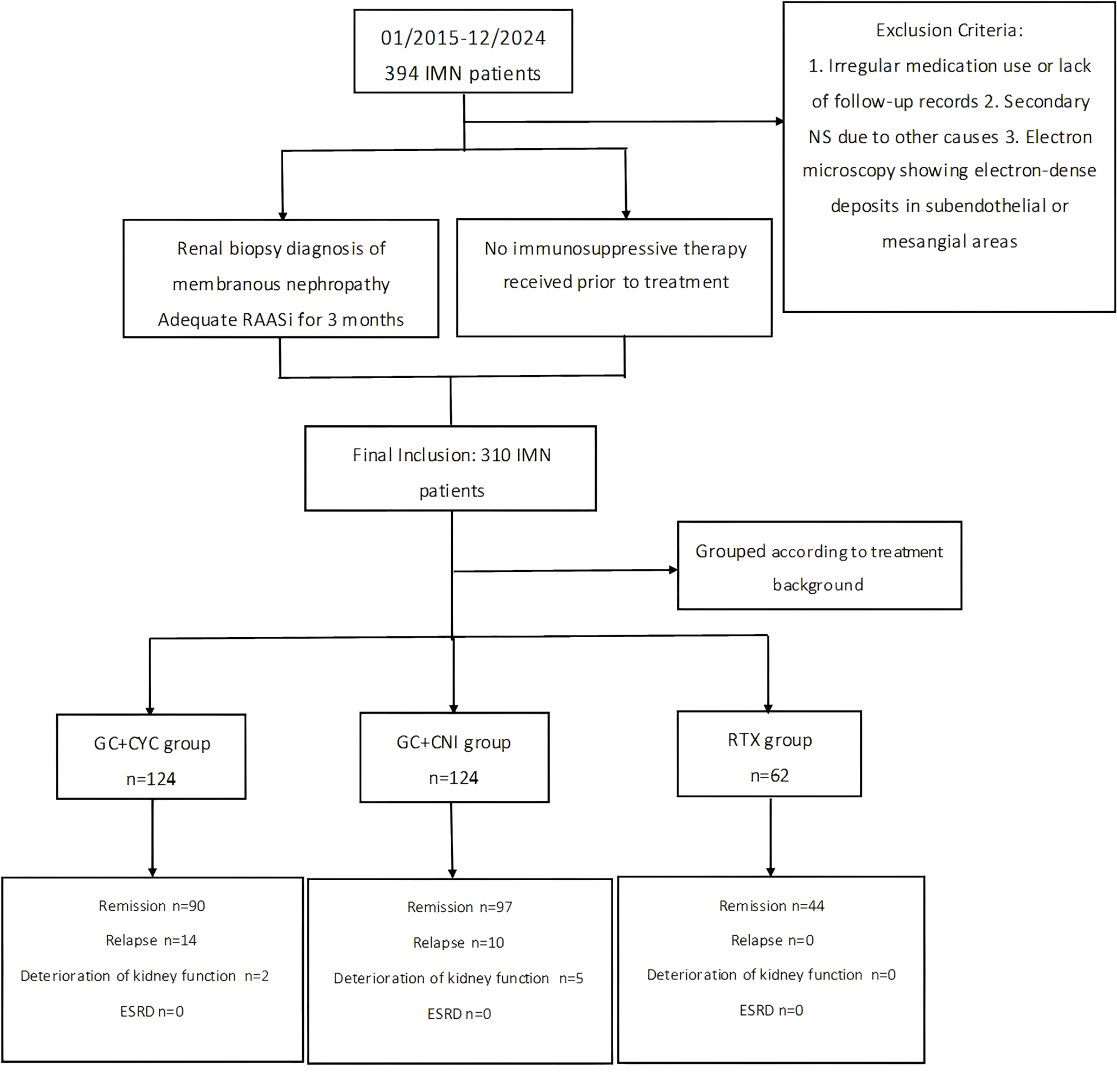
Flowchart of IMN patients receiving three treatment regimens.

In this study, patients were divided into three groups based on their treatment regimens: RTX monotherapy group (hereafter referred to as the “RTX group”), glucocorticoid combined with cyclophosphamide group (“GC+CYC group”), and glucocorticoid combined with calcineurin inhibitor group (“GC+CNI group”). A total of 310 patients were included in the study: 62 in the RTX group, 124 in the GC+CYC group, and 124 in the GC+CNI group. Details are shown in **Figure 1**. The study was reviewed and approved by the Medical Ethics Committee of Shandong Provincial Hospital, affiliated with Shandong First Medical University (JNKJ: NO.2020-3028). All treatment regimens were agreed upon by the patients and their families, and informed consent was obtained.

**Figure 2:**
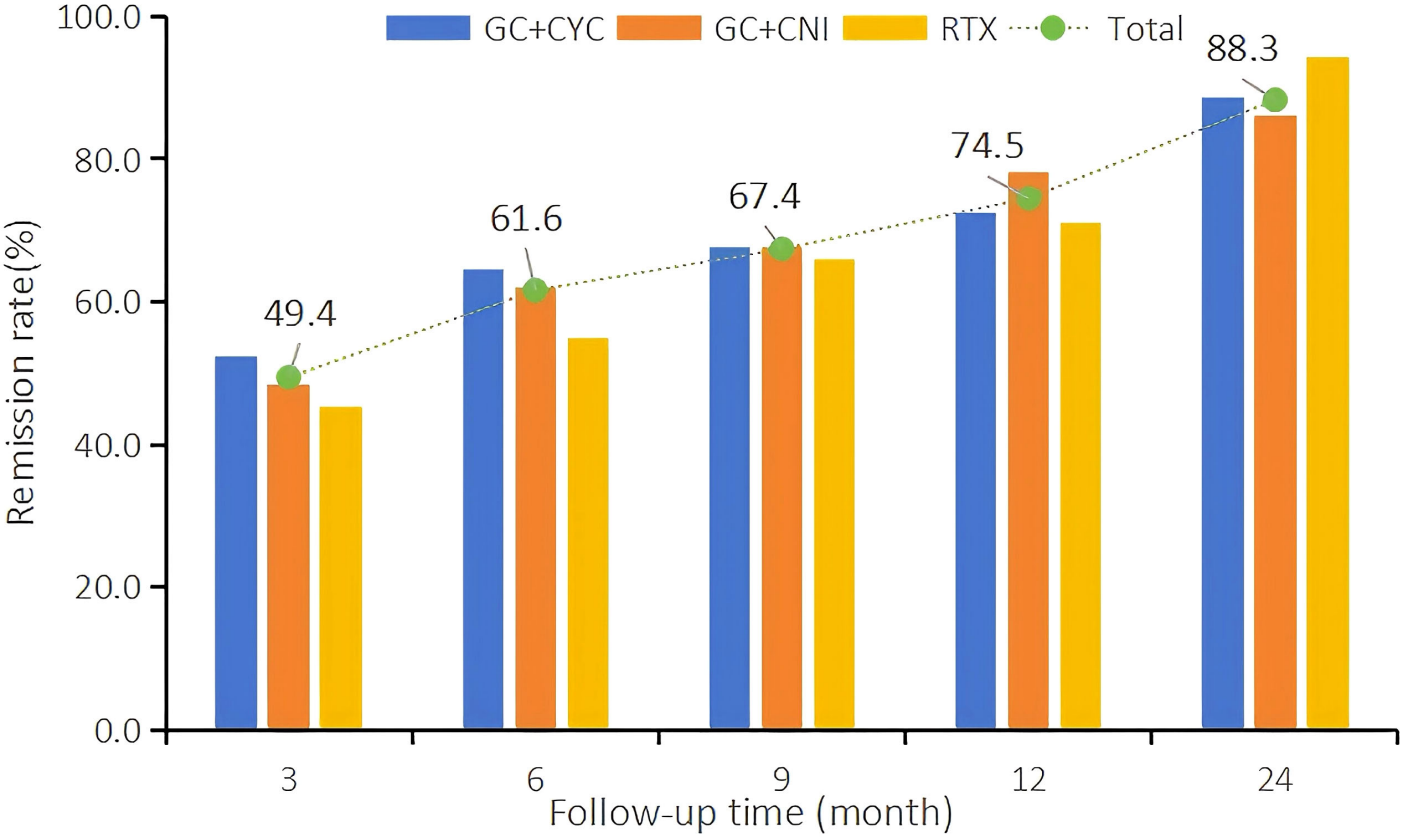
Trends in clinical remission rates during the 24-month follow-up period.

### Clinical and pathological data

General clinical data, including age and sex, of patients were collected. Laboratory data included complete blood count, liver and kidney function tests, blood lipid and glucose levels, urinalysis results, 24-hour proteinuria levels, anti-PLA2R antibody levels, and circulating B-cell counts for cellular immunity assessment. In this study, risk stratification of IMN patients was performed according to the standards of the 2021 KDIGO clinical practice guidelines for glomerulonephritis. The estimated glomerular filtration rate (eGFR) was calculated using the modified diet in renal disease formula: eGFR (mL/min/1.73 m²)=186 × [Scr (μmol/L)/88.4] − 1.154 × age − 0.203. For female patients, the result was further multiplied by a correction factor of 0.742.

Renal tissue obtained through biopsy from all patients was examined using light microscopy, immunofluorescence, and electron microscopy. Based on the electron microscopy findings, membranous nephropathy (MN) was classified into four stages (I–IV) according to the Ehrenreich–Churg staging system. When two stages were present in a patient’s pathology results, the higher stage was considered the final pathological stage.

### Treatment regimens and follow-up

CYC Administration Regimen: CYC was administered intravenously at a dosage of 12–15 mg/kg per month (approximately 0.6–1.2 g) continuously for at least 6 months. The regimen aims for a cumulative dose of no less than 4 g, with a target cumulative dose of 6–10 g. All patients received adequate doses of prednisone acetate in combination. The initial dosage was 1 mg/kg/day and was maintained for 8 weeks. Thereafter, the prednisone dosage was reduced by 5 mg every 2 weeks and then maintained at 10 mg/day. The total treatment duration was at least 6 months.

Tacrolimus (TAC) Administration Regimen: TAC was initially administered orally at a dosage of 0.05–0.1 mg/kg/day, with the blood trough concentration maintained at 5–10 ng/mL. The treatment duration was at least 6 months.

Cyclosporine Administration Regimen: Cyclosporine was initially administered orally at a dosage of 3–5 mg/kg/day, with the blood trough concentration maintained at 100–200 ng/mL. The treatment duration was at least 6 months. All patients receiving CNIs also received prednisone acetate at an initial dosage of 0.3–0.5 mg/kg/day. After 8 weeks of treatment, the prednisone dosage was reduced by 5 mg every 2–4 weeks and then maintained at 10 mg/day.

RTX Regimens: Two administration regimens were used. The first regimen involved intravenous administration of RTX at 375 mg/m² once weekly for 4 consecutive weeks (1 treatment cycle). The second regimen involved intravenous administration of RTX at 1 g per dose, given consecutively twice with a 2-week interval (1 treatment cycle). B-cell depletion was defined as an absolute circulating B-cell count of <5 cells/mm³ in the bloodstream.

All patients were followed up every 3 months. At each follow-up, laboratory tests were conducted, including complete blood count, liver and kidney function tests, lipid profile, blood glucose, urinalysis, 24-hour proteinuria quantification, and anti-PLA2R antibody levels. Patients in the CNI group underwent monitoring of blood drug concentrations, while those in the RTX group were monitored for circulating B-cell counts. Decisions regarding additional RTX administration were based on B-cell counts, anti-PLA2R antibody levels, and proteinuria remission status at 3–6 months after treatment initiation. All adverse events related to the treatment regimen were recorded during the follow-up period.

Follow-up assessments were conducted every 3 months before and after treatment to monitor and record complications and remission status. The primary endpoint was clinical remission at 12 months. Secondary endpoints, evaluated at 24 months, included clinical remission rate, relapse rate, and safety and occurrence of adverse events following treatment.

### Treatment effectiveness evaluation and renal outcomes

To evaluate treatment effectiveness, complete remission was defined as a 24-hour proteinuria level of <0.3 g, with stable kidney function (eGFR ≥45 mL/min/1.73 m²). Partial remission was defined as a reduction of at least 50% in 24-hour proteinuria levels from baseline, with proteinuria levels between 0.3 and 3.5 g, and stable kidney function (eGFR ≥45 mL/min/1.73 m²). Non-responders were defined as patients with <25% reduction in 24-hour proteinuria from baseline, indicating no clinical remission. Relapse was defined as a recurrence of 24-hour proteinuria >3.5 g in patients who had previously achieved complete or partial remission. The primary endpoint for renal outcomes was deterioration of kidney function or occurrence of ESRD. Deterioration of kidney function was defined as a post-treatment serum creatinine level >133 μmol/L or a sustained doubling of serum creatinine for > 3 months. ESRD was defined as a glomerular filtration rate <15 mL/min/1.73 m² during the follow-up period, at initiation of dialysis, or at the time of kidney transplantation. Serious adverse events were defined as conditions such as stroke, myocardial infarction, severe pulmonary infection, or pulmonary embolism that result in patient death or require hospitalization due to treatment-related adverse events.

### Statistical methods

All data were analyzed using SPSS software version 26.0. For continuous variables that followed a normal distribution, data are presented as mean ± standard deviation, and comparisons between two groups were made using the *t*-test. For continuous variables that did not follow a normal distribution, data are presented as median (interquartile range), and comparisons between two groups were made using the rank-sum test. For comparisons among three or more groups of continuous variables, one-way analysis of variance was used. Categorical variables are presented as frequencies, and comparisons between groups were made using the chi-square (χ²) test. All statistical tests were two-tailed, with a significance level of 0.05. A *P* value < 0.05 was considered statistically significant, and *P* < 0.01 was considered highly significant. Logistic regression analysis was conducted to identify potential risk or protective factors associated with treatment response, based on clinical relevance. For this analysis, statistical significance was defined as *P* < 0.10.

## Results

### Baseline data

**Figure 3:**
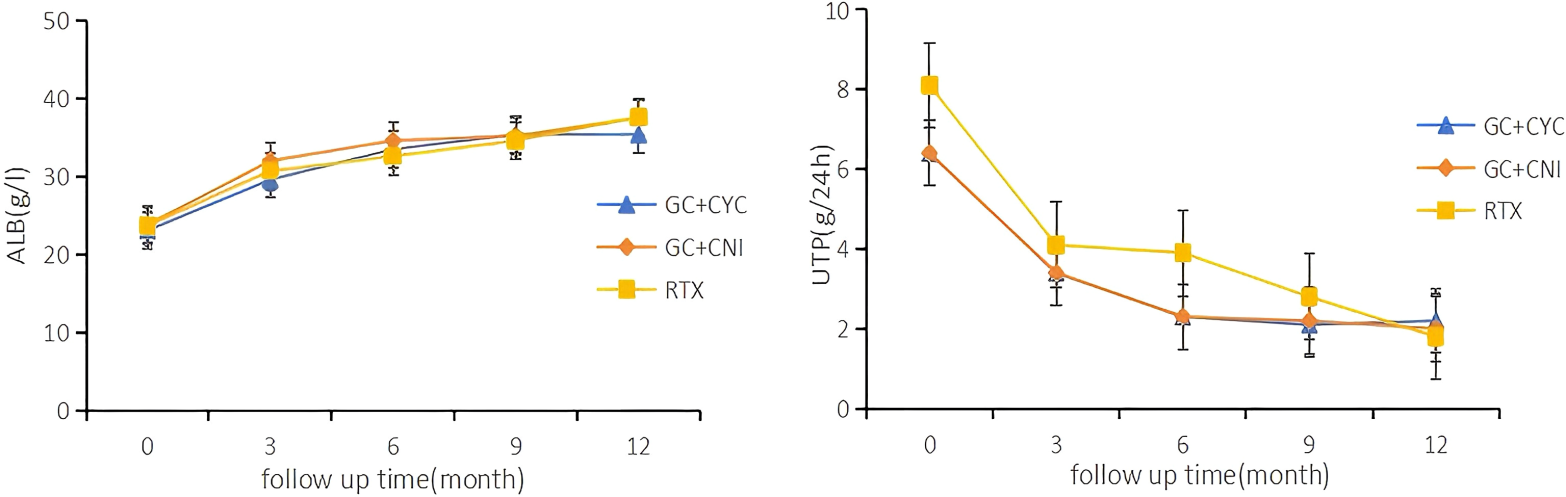
Serial levels of albumin and proteinuria after different treatments in patients who had been followed up for 12 months. Each point shows the mean of the variable.

A total of 310 patients with IMN were included in this study. Among them, 62 patients were included in the RTX group, 124 in the GC+CYC group, and 124 in the GC+CNI group. The median age of patients was 48.0 years (35.7, 57.0), with 213 male and 97 female patients. Before treatment, the median 24-hour proteinuria level for all patients was 5.8 (4.3, 8.3) g/24 h, the median serum albumin level was 23.7 (20.0, 27.0) g/L, and the median serum creatinine level was 76.0 (69.0, 79.0) μmol/L. The median eGFR was 101.0 (94.4, 113.3) mL/min/1.73 m², and the median anti-PLA2R antibody level was 71.0 (15.8, 220.8) U/mL. A total of 202 patients (65.2%) tested positive for anti-PLA2R antibodies (>20 U/mL). Patients in the RTX group were older than those in the GC+CYC group and GC+CNI group [50.5 (38.5, 57.7) vs. 49.0 (42.0, 58.0) vs. 45.5 (32.0, 55.0) years, *P* = 0.009]. The RTX group also had significantly higher proteinuria levels than those in the GC+CYC group and GC+CNI group[7.2 (5.5, 10.7) vs. 5.4 (4.3, 8.0) vs. 5.4 (3.9, 7.6) g/24 h, *P* = 0.006] and the highest number of patients positive for anti-PLA2R antibodies [42 (67.7%) vs. 78 (62.9%) vs. 82 (66.1%), *P* = 0.029]. In the RTX group, a larger proportion of patients were stratified as high-risk [25 (40.3%) vs. 43 (34.7%) vs. 45 (36.3%), *P* = 0.752], though this difference was not statistically significant. No significant differences were noted among the three groups in terms of anti-PLA2R antibody levels, albumin levels, or IgG levels. See **Table 1** and **Figure 3** for details.

**Table 1:** Baseline characteristics of IMN patients included in this study.

Characteristic	Total (n=310)	RTX (n=62)	GC+CYC (n=124)	GC+CNI (n=124)	*P*
Male sex, n (%)	213 (68.7)	41 (66.1)	90 (72.5)	82 (66.1)	0.501
Age (years)	48.0 (36.7, 57.0)	50.5 (38.5, 57.7)	49.0 (42.0, 58.0)	45.5 (32.0, 55.0)	**0.009**
Urine RBC/uL	6.8 (3.6, 17.3)	19.7 (7.7, 49.7)	5.4 (3.6, 9.5)	6.1 (3.2, 18.1)	**0.006**
Proteinuria (g/24 h)	5.8 (4.3, 8.3)	7.2 (5.5, 10.7)	5.4 (4.3, 8.0)	5.4 (3.9, 7.6)	**0.001**
WBC (×10^9^/L)	6.5 (5.3, 8.0)	6.6 (5.7, 8.1)	6.6 (5.1, 8.8)	6.3 (5.3, 7.7)	0.539
Hemoglobin (g/L)	141.0 (127.0, 151.0)	137.0 (125.0, 148.0)	140.0 (125.0, 150.0)	142.0 (129.0, 156.0)	0.120
Platelet (×10^9^/L)	259.0 (223.0, 307.5)	284.0 (219.0, 324.0)	259.0 (219.0, 297.0)	255.0 (225.0, 294.0)	0.389
AST (u/L)	21.0 (18.0, 26.0)	21.0 (16.0, 27.0)	22.0 (17.0, 27.0)	21.0 (18.0, 25.0)	0.850
ALT (u/L)	19.0 (14.0, 27.0)	19.0 (14.0, 24.0)	20.5 (15.0, 27.2)	17.0 (13.0, 27.0)	0.258
Total protein (g/L)	47.1 ± 6.6	47.6 ± 6.1	45.9 ± 6.6	48.1 ± 6.8	**0.024**
Albumin (g/L)	23.7 (20.0, 27.0)	23.9 (20.5, 27.0)	23.1 (19.8, 26.4)	24.0 (20.4, 27.8)	0.342
Globulin (g/L)	23.5 ± 3.8	23.9 ± 3.0	22.6 ± 3.8	24.2 ± 4.0	**0.004**
BUN (mmol/L)	5.1 (4.1, 6.5)	5.3 (4.3, 7.1)	5.4 (4.3, 6.6)	4.8 (3.9, 6.0)	**0.023**
Serum creatinine (μmol/L)^a^	76.0 (69.0, 79.0)	67.3 (53.2, 81.2)	76.4 (72.1, 79.0)	76.5 (70.8, 80.0)	**0.000**
eGFR (mL/min/1.73 m^2^)	101.0 (94.4, 113.3)	109.0 (96.7, 120.0)	99.5 (94.7, 106.9)	100.2 (93.8, 113.4)	0.099
Cholesterol (mmol/L)	8.5 ± 2.4	8.2 ± 2.7	8.5 ± 2.2	8.7 ± 2.6	0.452
IgG (g/L)^b^	5.5 ± 2.2	5.6 ± 2.3	5.3 ± 1.8	5.6 ± 2.4	0.523
Anti-PLA2R antibodies (U/mL)	71.0 (15.8, 220.8)	54.0 (13.6, 146.4)	73.2 (15.2, 267.5)	81.4 (16.7, 222.2)	0.245
Anti-PLA2R antibody positivity, n (%)	202 (65.2)	42 (67.7)	78 (62.9)	82 (66.1)	**0.029**
Low-risk, n (%)	0	0	0	0	0
Intermediate-risk, n (%)	197 (63.5)	37 (59.7)	81 (65.3)	79 (63.7)	0.752
High-risk, n (%)	113 (36.5)	25 (40.3)	43 (34.7)	45 (36.3)	0.752

Values are presented as number (%), median (interquartile range), or mean ± SD.

WBC, white blood cell; AST, glutamic oxaloacetic transaminase; ALT, glutamic pyruvic transaminase; BUN, blood urea nitrogen; eGFR, estimated glomerular filtration rate; IgG, immunoglobulin G; PLA2R, phospholipase A2 receptor.

a. eGFR is calculated according to the Chronic Kidney Disease Epidemiology Collaboration equation.

b. Anti-PLA2R positivity is defined by a value>20 RU/ml. Values in bold represent P<0.05

### Pathological data

In the RTX group, patients with pathological stage II had a higher remission rate [29 (85%) vs. 28 (69.6%) vs. 5 (50%), *P* = 0.346] than those with pathological stages I and III, though the difference was not statistically significant. In the GC+CYC group, patients with pathological stage III had a higher remission rate [2 (100%) vs. 78 (84.6%) vs. 44 (86.4%), *P* = 0.813] than those with pathological stages I and II, but again, the difference was not statistically significant. In the GC+CNI group, which included only patients with pathological stages I and II, the remission rate was slightly higher for those with pathological stage I than for those with pathological stage II [98 (87.1%) vs. 26 (82.6%)], although the difference was not statistically significant (*P* = 0.816). See **Table 2** for details.

**Table 2:** Pathological stage and clinical response rate in three treatment groups.

Group	No. of patients with remission/total no. (%) at 12 months			P
	Stage I	Stage II	Stage III	
RTX	28 (69.6)	29 (85.0)	5 (50)	0.346
GC+CYC	78 (84.6)	44 (86.4)	2 (100)	0.813
GC+CNI	98 (87.1)	26 (82.6)		0.816

### Treatment effectiveness evaluation

All patients in this study completed at least 12 months of follow-up. During the 12-month follow-up, a significant reduction was observed in the 24-hour proteinuria levels for all patients, while serum albumin levels exhibited an upward trend. These changes are detailed in **Figure 3**. At the 12-month follow-up, serum albumin levels increased from 23.7 (20.0, 27.0) g/L to 38.0 (34.5, 41.0) g/L. The RTX group showed higher albumin levels [39.0 (35.3, 41.8) vs. 37.0 (32.2, 40.0) vs. 38.7 (35.6, 42.0) g/L, *P* = 0.004] those in the GC+CYC group and GC+CNI group. Anti-PLA2R antibody levels decreased from 71.0 (15.8, 220.8) U/mL to 2.0 (2.0, 15.7) U/mL, with statistically significant differences observed among the three groups. The RTX group demonstrated a more pronounced reduction [2.0 (2.0, 2.1) vs. 6.4 (2.0, 54.6) vs. 6.6 (2.0, 70.4) U/mL, *P* = 0.000], resulting in achieving complete immunologic clearance. The 24-hour proteinuria level decreased from 5.8 (4.3, 8.3) g/24 h to 1.0 (0.2, 3.1) g/24 h; however, the differences among the three groups were not statistically significant. See **Table 3** for details.

**Table 3:** Clinical characteristics of IMN patients after 12 months of rituximab treatment.

Clinical characteristics	Total	GC+CYC	GC+CNI	RTX	*P*
Proteinuria (g/24 h)	1.0 (0.2, 3.1)	0.9 (0.2, 3.6)	1.1 (0.2, 3.1)	0.9 (0.3, 2.4)	0.936
Albumin (g/L)	38.0 (34.5, 41.0)	37.0 (32.2, 40.0)	38.7 (35.6, 42.0)	39.0 (35.3, 41.8)	**0.004**
Serum creatinine (μmol/L)^a^	66.0 (56.7, 75.3)	63.0 (55.9, 71.1)	68.0 (61.9, 80.2)	65.4 (53.0, 81.2)	**0.000**
eGFR (mL/min/1.73 m^2^)	112.3 (93.9, 128.3)	117.6 (102.5, 135.8)	110.0 (89.5, 121.5)		**0.001**
BUN (mmol/L)	5.6 (4.8, 7.1)	5.4 (4.7, 6.6)	5.7 (5.0, 7.4)		0.091
Anti-PLA2R antibodies (U/mL)	2.0 (2.0, 15.7)	6.4 (2.0, 54.6)	6.6 (2.0, 70.4)	2.0 (2.0, 2.1)	**0.000**

eGFR, estimated glomerular filtration rate; BUN, blood urea nitrogen; PLA2R, phospholipase A2 receptor.

a. eGFR is calculated according to the Chronic Kidney Disease Epidemiology Collaboration equation. Values in bold represent P<0.05

At the 12th month of treatment across the three treatment regimens, a total of 231 IMN patients (74.5%) achieved clinical remission; among these, 118 (38.1%) achieved complete remission. In the RTX group, 44 patients (71.0%) achieved clinical remission, and of these, 18 (29.0%) achieved complete remission. In the GC+CYC group, 90 patients (72.6%) achieved clinical remission, with 48 (38.7%) achieving complete remission. In the GC+CNI group, 97 patients (78.2%) achieved clinical remission, and of these, 52 (41.9%) achieved complete remission. At the 24th month of treatment, a total of 233 patients (88.3%) across the three regimens achieved clinical remission, and of these, 109 (41.3%) achieved complete remission. In the RTX group, 33 patients (94.3%) achieved clinical remission, with 18 (51.4%) achieving complete remission. In the GC+CYC group, 102 patients (88.7%) achieved clinical remission, and of these, 44 (38.3%) achieved complete remission. In the GC+CNI group, 98 patients (86.0%) achieved clinical remission, with 47 (41.2%) achieving complete remission. See **Table 4** and **Figure 2** for details.

**Table 4:** Complete remission or composite (complete or partial remission) from 3 to 24 months based on intention-to-treat analysis.

Study Time Points	No. of patients with remission of total no. (%)				P
	Total	RTX	GC+CYC	GC+CNI	
Complete remission					
3 months	12/310 (3.9)	1/62 (1.6)	3/124 (2.4)	8/124 (6.5)	0.125
6 months	44/310 (14.2)	5/62 (8.1)	18/124 (14.5)	21/124 (16.9)	0.261
9 months	81/310 (26.1)	12/62 (19.4)	33/124 (26.6)	36/124 (29.0)	0.362
12 months	118/310 (38.1)	18/62 (29.0)	48/124 (38.7)	52/124 (41.9)	0.228
24 months	109/264 (41.3)	18/35 (51.4)	44/115 (38.3)	47/114 (41.2)	**0.000**
Complete or partial remission					
3 months	153/310 (49.4)	28/62 (45.2)	65/124 (52.4)	60/124 (48.4)	0.622
6 months	191/310 (61.6)	34/62 (54.8)	80/124 (64.5)	77/124 (62.1)	0.437
9 months	209/310 (67.4)	41/62 (66.1)	84/124 (67.7)	84/124 (67.7)	0.971
12 months	231/310 (74.5)	44/62 (71.0)	90/124 (72.6)	97/124 (78.2)	0.460
24 months	233/264 (88.3)	33/35 (94.3)	102/115 (88.7)	98/114 (86.0)	**0.002**

The primary outcome is complete remission at 12 months. The remission rate is 74.5% (231/310) in total. The RTX treatment regimen has a higher remission rate than those in the GC+CYC group and GC+CNI group at 24 months (94.3% vs 88.7% vs 86.0%, *P* = 0.002).

Values in bold represent *P* < 0.05.

During follow-up, the clinical remission rates in all groups increased gradually with longer follow-up. At 12 months of follow-up, the clinical and complete remission rates in the RTX group were slightly lower than those in the other two groups, but the differences were not statistically significant. At 24 months of treatment, the clinical (94.3%) and complete remission rates (51.4%) in the RTX group were significantly higher than those in the GC+CYC group (88.7% and 38.3%, respectively) and GC+CNI group (86.0% and 41.2%, respectively).

### Risk factor analysis for different treatment regimens of IMN

Univariate logistic regression analysis indicated that anti-PLA2R antibody titer (OR = 0.998, *P* = 0.001) and serum creatinine level [odds ratio (OR)=0.981, *P* = 0.081] were risk factors for non-remission, while globulin level (OR = 1.083, *P* = 0.052) was a protective factor. Specifically, anti-PLA2R antibody titer (OR = 0.998, *P* = 0.016) was confirmed as an independent risk factor for non-remission. See **Table 5** for details.

**Table 5:** Risk factors for no-remission of IMN patients (logistic regression).

Characteristics	Univariate analysis		Multivariate analysis	
	OR (95% CI)	*P* value	OR (95% CI)	*P*
Male sex	1.752 (0.855, 3.590)	0.125	0.429 (0.892, 6.615)	0.082
Age (years)	0.989 (0.965, 1.014)	0.347	0.963 (0.924, 1.004)	0.076
Proteinuria (g/24 h)	0.964 (0.881, 1.055)	0.425	1.033 (0.908, 1.176)	0.618
Urine RBC/uL	0.996 (0.990, 1.003)	0.288	0.997 (0.984, 1.010)	0.656
ALT	1.016 (0.990, 1.042)	0.228	1.016 (0.969,1.066)	0.514
AST	1.013 (0.984, 1.043)	0.376	1.069 (0.986, 1.160)	0.106
Total protein (g/L)	1.033 (0.986, 1.082)	0.175	1.051 (0.763, 1.448)	0.760
Albumin (g/L)	1.008 (0.940, 1.081)	0.817	0.875 (0.618, 1.239)	0.452
Globulin (g/L)	1.083 (0.999, 1.174)	**0.052**	1.006 (0.723, 1.401)	0.969
Cholesterol (mmol/L)	1.041 (0.913, 1.186)	0.550	1.058 (0.873, 1.282)	0.563
Serum creatinine (μmol/L)	0.981 (0.961, 1.002)	**0.081**	0.974 (0.948, 1.000)	**0.053**
eGFR (mL/min/1.73 m^2^)^a^	1.000 (0.984, 1.016)	0.996	0.980 (0.958, 1.002)	0.068
BUN (mmol/L)	0.926 (0.844, 1.016)	0.105	0.927 (0.836, 1.028)	0.152
IgG (g/L)	1.088 (0.933, 1.269)	0.281	1.064 (0.802, 1.413)	0.665
Anti-PLA2R antibodies (U/mL)^b^	0.998 (0.997, 0.999)	**0.001**	0.998 (0.997, 1.000)	**0.016**

ALT, glutamic pyruvic transaminase; AST, glutamic oxaloacetic transaminase; eGFR, estimated glomerular filtration rate; BUN, blood urea nitrogen; IgG, immunoglobulin G; PLA2R, phospholipase A2 receptor.

a. eGFR is calculated according to the Chronic Kidney Disease Epidemiology Collaboration equation.

b. Anti-PLA2R positivity is defined by a value>20 RU/ml.

c. Bold values represents *P* < 0.1.

### Safety analysis

During the follow-up period, the incidence of adverse events was significantly lower in the RTX group than in the GC+CYC and GC+CNI groups (17 [27.4%] vs. 71 [57.3%] vs. 85 [68.5%], *P* = 0.000). The RTX group also had the lowest incidence of serious adverse events among the three groups. A total of seven patients experienced deterioration of kidney function—five cases in the GC+CNI group and two in the GC+CYC group. Among the serious adverse events, femoral head necrosis (3.2%) was more common in the GC+CYC group, while severe pneumonia was reported in all three groups. Importantly, no deaths resulting from these events were observed. Infusion reactions and arthralgia were more common in the RTX group. Infusion reactions presented as rash, mild cough, urticaria, rhinorrhea, and pruritus. The most common adverse reactions in the GC+CYC group were hair loss (8.9%), infection (8.1%), and liver damage (6.5%), while adverse reactions such as hypertension (12.9%) and hyperglycemia (12.1%) were common in the GC+CNI group. No patients experienced fatal adverse events such as malignancies or death during the study. See **Table 6** for details.

**Table 6:** Adverse events.

Events	RTX (n=62) patients, (n%)	GC+CYC (n=124) patients, (n%)	GC+CNI (n=124) patients, (n%)	*P*
Any adverse event Serious adverse event	17 (27.4)	71 (57.3)	85 (68.5)	0.000
Fatal	0	0	0	
Nonfatal	1 (1.6)	8 (6.5)	8 (6.5)	
Femoral head necrosis	0	4 (3.2)	1 (0.8)	
Severe pneumonia	1 (1.6)	2 (1.6)	2 (1.6)	
Deterioration of kidney function	0	2 (1.6)	5 (4.0)	
Nonserious adverse event	16 (25.8)	63 (50.8)	77 (62.1)	
Hyperglycemia	0	7 (5.6)	15 (12.1)	
Infection	2 (3.2)	10 (8.1)	9 (7.3)	
Hypertension	1 (1.6)	4 (3.2)	16 (12.9)	
Cerebral infarction	0	2 (1.6)	1 (0.8)	
Herpes zoster	0	5 (4.0)	2 (1.6)	
Leukopenia	0	7 (5.6)	3 (2.4)	
Arthralgia	4 (6.4)	3 (2.4)	4 (3.2)	
Hair loss	0	11 (8.9)	7 (5.6)	
Liver damage	0	8 (6.5)	6 (4.8)	
Gingival hyperplasia	0	0	6 (4.8)	
Infusion reactions*	7 (11.3)	0	0	
Lower extremity venous thrombosis	1 (1.6)	3 (2.4)	2 (1.6)	
Cardiac arrhythmia	1 (1.6)	1 (0.8)	3 (2.4)	
Diarrhea	0	2 (1.6)	3 (2.4)	

*Infusion reactions include rash, slight cough, rhinorrhea, urticaria, pruritus, bronchial wheezing, erythema, and dysphoria.

## Discussion

The 2021 KDIGO guidelines (6) recommend aggressive immunosuppressive therapy for MN patients at risk of progression or those stratified as intermediate-risk or higher-risk. Commonly used immunosuppressive treatment regimens include GC combined with CYC or CNI, as well as biologics such as RTX. To provide personalized and precise treatment regimens for IMN patients, we designed this study to retrospectively analyze the treatment effectiveness and safety of these three treatment regimens. After 12 months of treatment with the three regimens, most patients achieved clinical remission. No significant differences were noted in clinical remission rates among the three groups. However, the RTX group had significantly lower relapse rates and fewer adverse events than the other two groups, and no cases of kidney function deterioration were observed. With the follow-up period extended to 24 months, the clinical and complete remission rates in the RTX group were superior to those in the other two groups.

In this study, 71.0% of patients in the RTX group achieved clinical remission at 12 months. This remission rate is slightly higher than the 60% reported in the MENTOR (9) study, the 62% in the RI-CYCLO study (14), and the 64.9% in the GEMRITUX study (15). The primary reason for the higher remission rate is the strict adherence to the standard dosing regimen of RTX in the first month, either 375 mg/m² × 4 doses or 1 g × 2 doses. Additionally, 24.2% of patients received a cumulative RTX dose of over 3.0 g, and 67.7% of patients received an additional RTX dose within 6 months. This rational supplementation ensured complete immunological remissions of anti-PLA2R antibodies during the treatment cycle [2.0 vs. 6.4 vs. 6.6 U/mL, P = 0.000], resulting in better clinical responses (16, 17). The overall remission rate in the GC+CYC group was 72.6%, consistent with the 73% at 12 months reported in the RI-CYCLO study. The GC+CNI group achieved an overall remission rate of 78.2% at 12 months, which is significantly higher than the 52% composite remission rate at 12 months reported in the MENTOR study. This difference can be attributed to two main factors: first, our study used CNI in combination with GC, unlike the MENTOR study, which used cyclosporine alone. GC can inhibit inflammatory mediators, suppress T and B lymphocytes, reduce antibody production, and act synergistically with CNIs to reduce proteinuria. The STARMEN study (18) showed that the overall and complete remission rates of tacrolimus combined with steroids were higher than those of tacrolimus monotherapy. Second, in our study, the GC+CNI group had lower baseline proteinuria [5.4 (3.9, 7.6) g/24 h] and anti-PLA2R antibody levels [81.4 (16.7, 222.2) U/mL] than the cyclosporine group in the MENTOR study, which had a proteinuria level of 8.9 (6.7, 12.9) g/24 h and an anti-PLA2R antibody level of 413 (206, 961) U/mL.

Compared to the GC+CYC and GC+CNI groups, the RTX group did not show a significant advantage in overall remission or complete remission rates at 12 months. This may be related to the fact that patients in the RTX group had higher 24-hour proteinuria levels, were stratified as high-risk, and included a larger proportion of older patients. However, as the treatment duration extended, both the composite and complete remission rates in the RTX group increased gradually, highlighting the advantages of RTX treatment. Additionally, the relapse rate was lower. The analysis suggests that RTX achieves immunological remission by depleting CD20-positive B cells and reducing antibody production, ultimately resulting in sustained clinical remission. At 12 months, the RTX group showed a greater reduction in anti-PLA2R antibody titer, decreasing from a baseline of 54.0 (13.6, 146.4) U/mL to 2.0 (2.0, 2.1) U/mL. This 96.3% reduction was greater than the 91.3% and 91.9% reductions observed in the other two groups. Furthermore, at 24 months, the anti-PLA2R antibody titer remained low at 2.0 (2.0, 3.3) U/mL. During the treatment period, the anti-PLA2R antibody titer decreased in parallel with reductions in CD19+ B-cell counts. These findings support the effectiveness of RTX in eliminating anti-PLA2R antibodies and suggests its importance in achieving clinical remission. Several studies currently define immunological remission as a PLA2R antibody titer below 2 RU/mL, with immunological remission occurring prior to proteinuria remission (16, 19, 20). The anti-PLA2R antibody titer is closely related to disease severity, treatment effectiveness, and prognosis (21). Piero Ruggenenti (22)’s study shows that clearance of anti-PLA2R antibodies at 6 months significantly increases the likelihood of achieving composite endpoints or complete remission. Moreover, lower antibody titers predict higher remission rates and shorter remission times. This may explain why the remission rates in the RTX group in our study increased with longer treatment durations.

We conducted a logistic regression analysis of factors that might influence clinical remission and found that the anti-PLA2R antibody titer and creatinine level are risk factors for non-remission in IMN patients, while the globulin level is a protective factor. The identification of the anti-PLA2R antibody titer as an independent risk factor for clinical non-remission in IMN patients is consistent with previous research (8).

Compared to the regular monthly intravenous injection of CYC and twice-daily oral administration of CNIs, intravenous infusion of RTX can reduce the frequency of patient follow-ups and hospitalization rates. This reduction leads to better adherence. From an economic perspective, the initial treatment cost of RTX may be higher; however, when considering expenses related to complications and relapse, as well as its advantage in maintaining long-term clinical remission, the overall cost of RTX decreases over time (23).The economic advantage of RTX is reflected in the overall costs over the entire treatment cycle and disease management. Its core benefits lie in its low relapse rate and favorable adverse effect profile, which significantly reduce the costs associated with managing adverse events, thereby saving long-term medical expenses across multiple aspects. The CYC regimen, while having a low initial cost, carries a substantial burden in terms of potential adverse event management. CNIs entail a moderate initial cost, but the high relapse rate and the need for therapeutic drug monitoring contribute to an increase in total costs.

In this study, adverse events were common across all three groups. The RTX group had significantly lower rates of all adverse events (27.4%) and serious adverse events (1.6%) compared to the other two groups. Common infusion-related reactions typically occurred during the first infusion and could be alleviated by reducing the infusion rate; consequently, no patients discontinued or adjusted their treatment regimens due to these reactions. The incidence rate (11.3%) was also lower than reported in related studies (24). This lower incidence may be attributed to our routine use of anti-allergy medications (dexamethasone, methylprednisolone sodium succinate, and promethazine hydrochloride) and the practice of extending infusion times during RTX administration (25, 26). Among serious adverse events, deterioration of kidney function was common and occurred primarily in the GC+CNI group in this study. The frequent occurrence of such deterioration in the GC+CNI group is consistent with the findings of previous research, which demonstrates that nephrotoxicity is a common side effect of CNIs (27, 28). The deterioration of kidney function in the GC+CNI group was related to excessively high CNI concentrations and failure to promptly adjust medication doses. In the GC+CYC group, there were two such cases, likely due to disease progression caused by unresolved proteinuria. No cases of deterioration of kidney function were observed in the RTX group. This study further confirms the safety of RTX in treating IMN.

This study has some limitations. First, as a retrospective study, patient selection bias is inevitable. In this study, there were differences in certain baseline characteristics among the three groups, particularly a significant difference in 24-hour proteinuria levels. This confirms the presence of selection bias. Second, due to the retrospective nature of the study, follow-up data could not be obtained for patients in the three treatment groups at the same time points. This made it challenging to accurately collect the specific remission times for patients. Third, because the number of patients in the RTX group was relatively small, we could not perform 1:1 matched group analyses across the three groups. The small sample size in the RTX group may have contributed to the lack of statistically significant differences between groups. However, even if more RTX patients were included, it is possible that the study results would not change. Therefore, larger, multicenter, head-to-head, randomized controlled prospective studies are needed to compare the treatment effectiveness of different regimens and identify the most suitable patients for each treatment regimen.

In conclusion, RTX demonstrates non-inferior clinical remission at 12 months compared to CYC and CNIs. RTX also has lower relapse rates and better safety. These advantages in maintaining long-term clinical remission suggest that RTX can be considered an effective treatment regimen for MN patients at risk of progression.

A total of 310 IMN patients are included: 62 received rituximab as initial therapy, 124 patients received the GC+CYC regimen, and 124 patients received the GC+CNI regimen. During follow-up, 7 patients experienced deteriorating kidney function—2 from the GC+CYC group and 5 from the GC+CNI group. The condition in no patients progressed to ESRD.

## Data Availability

The original contributions presented in the study are included in the article/supplementary material, further inquiries can be directed to the corresponding author/s.
