# Correction: Effectiveness and safety of rituximab monotherapy versus conventional regimens for adult idiopathic membranous nephropathy: real-world retrospective study

**DOI:** 10.3389/fimmu.2026.1828377

**Published:** 2026-03-20

**Authors:** Frontiers Production Office

**Affiliations:** Frontiers Media SA, Lausanne, Switzerland

**Keywords:** idiopathic membranous nephropathy, treatment regimens, clinical remission rate, anti-PLA2R antibody, rituximab

The article type was erroneously given as “Review”. The correct article type is “Original Research”.

The **Data availability statement** was erroneously missing from the article, due to the incorrect article type. The correct **Data availability statement** is:

“The original contributions presented in the study are included in the article/supplementary material, further inquiries can be directed to the corresponding author/s.”

The **Ethics statement** was erroneously missing from the article, due to the incorrect article type. The correct **Ethics statement** is:

“The study was reviewed and approved by the Medical Ethics Committee of Shandong Provincial Hospital, affiliated with Shandong First Medical University (JNKJ: NO.2020-3028). All treatment regimens were agreed upon by the patients and their families, and written informed consent was obtained.”

The original version of this article has been updated.

